# Evaluation of apoptosis imaging biomarkers in a genetic model of cell death

**DOI:** 10.1186/s13550-019-0487-8

**Published:** 2019-02-19

**Authors:** Vessela Vassileva, Stephen M. Stribbling, Chris Barnes, Laurence Carroll, Marta Braga, Joel Abrahams, Kathrin Heinzmann, Caroline Haegeman, Marion MacFarlane, Kathryn L. Simpson, Caroline Dive, Jamie Honeychurch, Timothy M. Illidge, Eric O. Aboagye

**Affiliations:** 1 Cancer Imaging Centre, Department of Surgery and Cancer, Faculty of Medicine, Imperial College London, Hammersmith Hospital Campus, Du Cane Road, London, W12 0NN UK; 2 MRC Toxicology Unit, Hodgkin Building, Lancaster Road, Leicester, LE1 9HN UK; 3 Clinical and Experimental Pharmacology Group, Cancer Research UK Manchester Institute, The University of Manchester, Alderley Park, Manchester, SK10 4TG UK; 4 Targeted Therapy Group, Division of Cancer Sciences, Manchester Cancer Research Centre, Christie Hospital, Manchester Academic Health Sciences Centre, National Institute of Health Research Biomedical Research Centre, Manchester, UK

**Keywords:** Apoptosis, Caspases, Molecular imaging, ^18^F-ICMT-11, PET, Radiotracer, Death-switch

## Abstract

**Purpose:**

We have previously developed the caspase-based radiotracer, ^18^F-ICMT-11, for PET imaging to monitor treatment response. We further validated ^18^F-ICMT-11 specificity in a murine melanoma death-switch tumour model with conditional activation of caspase-3 induced by doxycycline.

**Methods:**

Caspase-3/7 activity and cellular uptake of ^18^F-ICMT-11, ^18^F-ML-10 and ^18^F-FDG were assessed in B16ova and B16ovaRevC3 cells after death-switch induction.

Death-switch induction was confirmed in vivo in xenograft tumours, and ^18^F-ICMT-11 and ^18^F-ML-10 biodistribution was assessed by *ex vivo* gamma counting of select tissues. PET imaging was performed with ^18^F-ICMT-11, ^18^F-ML-10 and ^18^F-FDG. Caspase-3 activation was confirmed by immunohistochemistry.

**Results:**

Significantly increased caspase-3/7 activity was observed only in B16ovaRevC3 cells after death-switch induction, accompanied by significantly increased ^18^F-ICMT-11 (*p* < 0.001) and ^18^F-ML-10 (*p* < 0.05) and decreased ^18^F-FDG (*p* < 0.001) uptake compared with controls.

B16ova and B16ovaRevC3 tumours had similar growth in vivo; however, B16ovaRevC3 growth was significantly reduced with death-switch induction (*p* < 0.01). Biodistribution studies showed significantly increased ^18^F-ICMT-11 tumour uptake following death-switch induction (*p* < 0.01), but not for ^18^F-ML-10. Tumour uptake of ^18^F-ICMT-11 was higher than that of ^18^F-ML-10 after death-switch induction. PET imaging studies showed that ^18^F-ICMT-11 can be used to detect apoptosis after death-switch induction, which was accompanied by significantly increased expression of cleaved caspase-3. ^18^F-FDG signal decreased in tumours after death-switch induction.

**Conclusions:**

We demonstrate that ^18^F-ICMT-11 can be used to detect caspase-3 activation in a death-switch tumour model, independent of the confounding effects of cancer therapeutics, thus confirming its specificity and supporting the development of this radiotracer for clinical use to monitor tumour apoptosis and therapy response.

**Electronic supplementary material:**

The online version of this article (10.1186/s13550-019-0487-8) contains supplementary material, which is available to authorized users.

## Introduction

Apoptosis, or programmed cell death, plays a crucial role during embryonic development, during differentiation, and in the maintenance of tissue homeostasis. Therefore, alterations in apoptotic signalling pathways occur in various disease pathologies; for example, excessive apoptosis is observed in ischaemia and transplant rejection [1], while insufficient apoptosis and the capacity to evade apoptosis are regarded as hallmarks of cancer [2, 3]. As cancer treatments often kill tumour cells through apoptosis, monitoring of this process can be useful in predicting clinical response to therapy, although it is appreciated that other mechanisms of cell death, including autophagy, necrosis, mitotic catastrophe and senescence can also occur [4, 5].

Apoptosis can be triggered through several signalling pathways and involves a family of cysteine-dependent aspartate-directed proteases, known as caspases [6, 7].

These are divided into two groups: initiator caspases (caspase-2, caspase-8, caspase-9 and caspase-10), and effector caspases (caspase-3, caspase-6 and caspase-7) [8]. Initiator caspases are activated in response to cellular stress, including radio- and chemo-therapy; these, in turn, cleave and activate executioner caspases, which orchestrate the demolition phase of the process, involving the degradation of key cellular structures and the subsequent disposal of the cell [8, 9]. Although there are several apoptosis-initiating signalling pathways (extrinsic, intrinsic and endoplasmic reticulum stress pathways), triggered by various stimuli, the execution phase of the process is mostly shared [4, 10] and involves the sequential activation of caspases. Caspase-3 plays a central role in the execution phase of apoptosis and is involved in the cleavage and activation of caspase-6 and caspase-7 [9, 11]. Therefore, caspase-3 is an attractive molecular target for imaging of apoptosis. Indeed, several molecules that bind to caspase-3 have been investigated for PET imaging of apoptosis, including small molecule inhibitors of the isatin sulphonamide class, which selectively bind to the active site of caspase-3 and caspase-7 [12–14]. Despite these promising developments, there are limited clinical examples of PET imaging of apoptosis.

We have developed and validated an isatin-based caspase-3/7-specific radiotracer, ^18^F-ICMT-11, for the in vivo assessment of tumour apoptosis and for monitoring response to cancer therapy by PET imaging, and have also shown favourable clinical dosimetry and safety profiles of ^18^F-ICMT-11 in a phase I trial [14–19]. Although several other apoptosis imaging probes targeting different processes in the apoptotic cascade have been developed, including annexin V, synaptotagmin I, ^18^F-ML-10, ^18^F-C-SNAT, ^18^F-fluorobenzyl triphenylphosphonium cation and ^18^F-CP18, no PET imaging agent has been approved for clinical use [20–27].

The present study aimed to investigate and further validate ^18^F-ICMT-11 as a caspase-3-specific, non-invasive imaging radiotracer in a death-switch syngeneic murine tumour model. This tumour model was generated by transfecting the ovalbumin-expressing mouse B16 melanoma cell line (B16ova) with a tetracycline-regulated reverse transcriptional transactivator, enabling the conditional induction of constitutively active caspase-3 (B16ovaRevC3) [28]. It has been shown that induction of this death-switch results in rapid and synchronous apoptosis both in vitro and in vivo [28]. Thus, we wanted to validate ^18^F-ICMT-11 as an apoptosis radiotracer in this genetic model of conditional caspase-3 activation, which offers an experimental system devoid of confounding drug effects, and the transcriptional evolution and translation of caspase-3 activity over time after doxycycline administration. Therefore, this model is a useful tool for the validation of ^18^F-ICMT-11. We also evaluated another apoptosis radiotracer, ^18^F-ML-10 [27], which is at a similar stage of development as ^18^F-ICMT-11 along the translational pathway, and ^18^F-FDG to assess tumour glucose metabolism and proliferation.

## Materials and methods

### Synthesis of radiotracers

Synthesis and radiolabelling of ^18^F-ICMT-11 and ^18^F-ML-10 were performed according to previously described protocols [29, 30]. ^18^F-ICMT-11 and ^18^F-ML-10 non-decay-corrected radio-chemical yields were 8–16% and 18–24% respectively. Radiochemical purity was > 98% at the end of synthesis for both radiotracers. ^18^F-FDG was obtained from Alliance Medical (London, UK).

### Cell culture

The murine melanoma B16ova and B16ovaRevC3 cell lines were kindly provided by Prof. Illidge (Manchester, UK). Both cell lines were maintained in an RPMI-1640 medium supplemented with 10% foetal calf serum, 2 mM l-glutamine, 100 U/ml penicillin and 100 μg/ml streptomycin (Invitrogen, UK), at 37 °C in a humidified atmosphere containing 5% CO_2_. The medium for B16ovaRevC3 cells was further supplemented with 500 μg/ml hygromycin B (Corning).

### In vitro induction and assessment of apoptosis

Apoptosis was assessed in B16ova and B16ovaRevC3 cells in response to doxycycline (doxycycline hyclate, Sigma) based on caspase activity using the Caspase-Glo-3/7 assay (Promega) according to the manufacturer’s protocol. Briefly, cells were exposed to doxycycline (0 or 2 μg/ml) for 1, 6, 24 or 48 h, and then incubated with Caspase-Glo reagent for 1 h. Luminescence was measured using a TopCount NXT microplate luminescence counter (PerkinElmer). Luminescence signal was normalized to protein content, which was determined using the BCA 96-well plate assay (Thermo Fisher Scientific). Experiments were performed in triplicate and data were expressed as fold-difference in caspase-3/7 activity (per mg of protein) from controls (mean ± SEM).

### In vitro tumour cell uptake of radiotracers

Cell uptake studies of the radiotracers were performed as previously described [17]. Briefly, B16ova or B16ovaRevC3 cells (1 × 10^5^ per well) were plated into six-well plates and incubated overnight. Cells were then exposed to doxycycline (0, 2 μg/ml) for 6 h (since caspase-3/7 activity was highest at this time point), and then incubated with 0.74 MBq/well of ^18^F-ICMT-11, ^18^F-ML-10 or ^18^F-FDG for 1 h at 37 °C in a humidified atmosphere containing 5% CO_2_. The medium was then removed, and cells were washed twice with PBS, prior to lysis in RIPA buffer (Thermo Fisher Scientific) for 15 min. Radioactivity in cell lysates was measured by gamma counting (Wizard 2480 Automatic Gamma Counter, Perkin Elmer). Protein content of the lysates was determined using the Pierce BCA assay (Thermo Fisher Scientific). Experiments were performed in triplicate, using six replicates per assay, and data were expressed as percent of total decay-corrected radioactivity per milligramme protein (mean ± SEM).

### In vivo tumour model generation and induction of the death-switch

All animal experiments were performed in accordance with the United Kingdom Home Office Guidance on the Operation of the Animals (Scientific Procedures) Act 1986 Amendment regulations 2012 and within the published National Cancer Research Institutes Guidelines for the welfare and use of animals in cancer research [31]. Studies were conducted under Project Licence Number PPL 70/8651. Tumour xenografts were established in female C57BL/6 mice (6–8 weeks old, Envigo) using B16ova or B16ovaRevC3 cells as previously described [28]. Briefly, 1 × 10^6^ tumour cells were injected subcutaneously into the neck region of the mice. Tumour dimensions were measured with callipers, and tumour growth was assessed based on tumour volume, calculated using the formula: volume = *π*/6 (length × width × height). Tumour growth was compared between B16ova and the genetically modified B16ovaRevC3 xenografts. To activate the death-switch in vivo, 10 days post tumour cell inoculation, doxycycline (2 mg/dose, p.o.) or water (vehicle-control) was administered once per day by oral gavage for 5 days to mice bearing B16ovaRevC3 xenografts (similar tumour volumes, *n* = 6 per group) and tumour volumes were measured for the treatment duration to show changes in tumour growth following death-switch induction. To ensure animal welfare, the weights of mice were also monitored to qualitatively assess possible adverse effects.

### Biodistribution of apoptotic radiotracers

Apoptotic radiotracer biodistribution (^18^F-ICMT-11 and ^18^F-ML-10) was evaluated in the B16ovaRevC3 tumour model, 24 h after vehicle-control or doxycycline administration to allow activation of the death-switch, as previously reported [28] and based on studies of doxycycline pharmacokinetics [32]. Briefly, mice received a single p.o. administration of vehicle-control or doxycycline (2 mg); 24 h later, mice were anaesthetized with isoflurane, and the lateral tail vein was cannulated for the i.v. administration of ^18^F-ICMT-11 or ^18^F-ML-10 (1.3 ± 0.2 MBq per mouse). Mice were allocated to two groups: (1) control and (2) doxycycline (*n* = 6 mice per group for each radiotracer). One hour after radiotracer administration, mice were sacrificed and select tissues were excised, and radioactivity was measured by *ex vivo* gamma counting in these select tissues (Wizard 2480 Automatic Gamma Counter, Perkin Elmer). Radiotracer biodistribution was calculated as percentage of injected dose per gram of tissue (%injected dose/g).

### PET imaging

PET imaging using the apoptotic radiotracers, ^18^F-ICMT-11 and ^18^F-ML-10, and the gold-standard ^18^F-FDG radiotracer was conducted in mice with B16ova and B16ovaRevC3 tumour xenografts 24 h after vehicle-control or doxycycline administration (as above for biodistribution studies), using parameters based on previous radiotracer distribution studies [14, 17, 33, 34]. Table 1 provides a summary of the radiotracers (specific activities, injected doses and volumes). Briefly, mice bearing B16ova or B16ovaRevC3 tumour xenografts received vehicle-control or a single dose of doxycycline (2 mg, p.o.); 24 h later, ^18^F-ICMT-11, ^18^F-ML-10 or ^18^F-FDG were administered and PET imaging was conducted. Mice were allocated to two groups: (1) control and (2) doxycycline (*n* = 6 mice per group) for each tumour model and for each radiotracer (^18^F-ICMT-11, ^18^F-ML-10 and ^18^F-FDG). An additional group (*n* = 6) for each tumour model was included to evaluate whether ^18^F-ICMT-11 can be used to image caspase-3 activation at an earlier time point, 6 h after doxycycline administration.

**Table 1:** Radiotracer specifications

Radiotracer	Specific activity	Injected dose	Injected volume
	(GBq/μmol ± SD)	(MBq ± SD)	(μl) ¥
^18^F-ICMT-11	155.6 ± 69.4	1.32 ± 0.15	25–190
^18^F-ML-10	107.8 ± 1.9	1.28 ± 0.19	25–200
^18^F-FDG	N/D ¶	1.26 ± 0.19	25–190

Dynamic PET scan started immediately after i.v. administration of radiotracers with acquisition time of 1 h

¶ Clinical grade as supplied by Alliance Medical

¥ The total injection volume was topped up to 200 μl with saline

Imaging was performed using the Genisys 4 Preclinical PET scanner (Sofie Biosciences, USA). Based on previous studies of radiotracer distribution, data were acquired over 30–60 min in list mode and corrected for decay. 3D ML-EM (60 iterations) reconstruction was performed and presented as summed 30–60-min frames (6x5min). Image visualization was performed using the Siemens Inveon Research Workplace software as previously described [17].

### Immunohistochemistry

Following PET imaging, tumour tissues were collected, fixed in formalin, embedded in paraffin, and sectioned. Immunohistochemistry for cleaved caspase-3 was performed on tumour sections to evaluate apoptosis. Briefly, after antigen retrieval, sections were incubated with a primary antibody for cleaved caspase-3 (Cell Signaling, 1:500) overnight at 4 °C, and visualized using biotin-conjugated secondary antibodies (Vectastain ABC Kit; Vector Labs) and 3, 3′-diaminobenzidine (DAB; Dako). Sections were then counterstained with haematoxylin and mounted with DPX (Sigma Aldrich).

Image acquisition of tumour sections was performed using the AxioScan digital slide scanner (Zeiss), and QuPath (Queen’s University Belfast, Northern Ireland) image analysis software was used for IHC quantification [35]. Briefly, regions of interest were defined to include viable tumour tissue, excluding necrotic areas. Cell-based analysis was performed with automated cell segmentation based on colour: haematoxylin in the blue and DAB in the red channel. To avoid artefacts, a threshold for minimum cell area and variance of haematoxylin staining was set. Positive cells were selected based on mean and maximum intensity of DAB. Data were generated by calculating the percentage of the DAB-reactive cells in relation to the total number of cells in the regions of interest.

### Statistical analyses

Data were plotted and analysed using Prism, version 7.0 (GraphPad Software) and expressed as mean ± standard deviation. Differences between the groups were assessed by unpaired Student’s and multiple *t* tests, ANOVA and Tukey’s or Sidak’s multiple comparisons tests. Results were considered statistically significant at a *P* value of 0.05 or less.

## Results

**Fig. 1:**
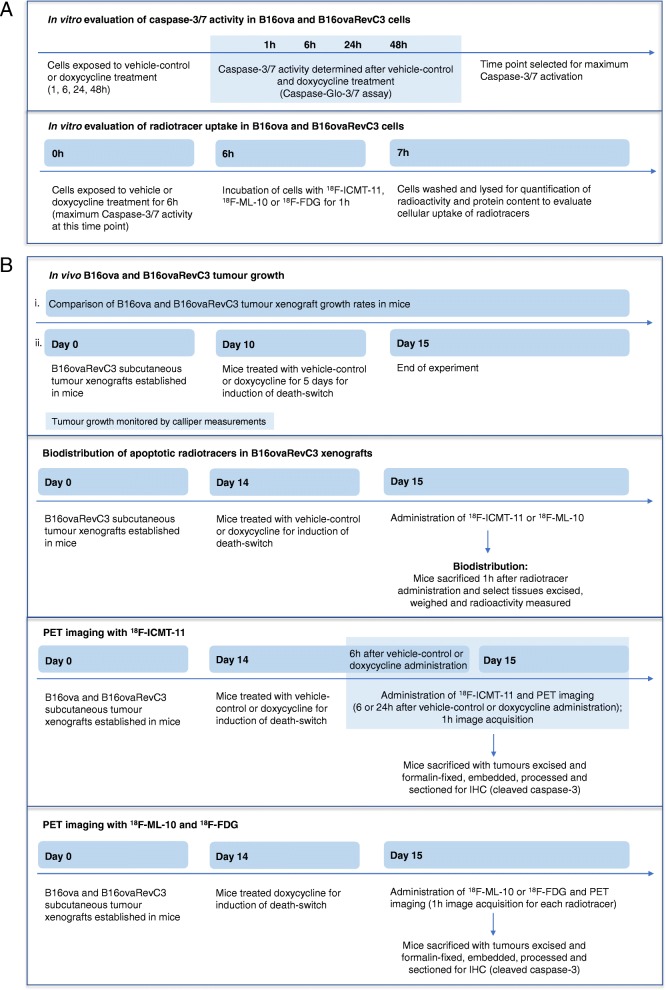
Experimental paradigm. Overview of **a** in vitro and **b** in vivo studies

We have outlined the overall experimental paradigm in Fig. 1, summarising the in vitro (Fig. 1a) and the in vivo (Fig. 1b) studies.

### In vitro caspase-3/7 activity

**Fig. 2:**
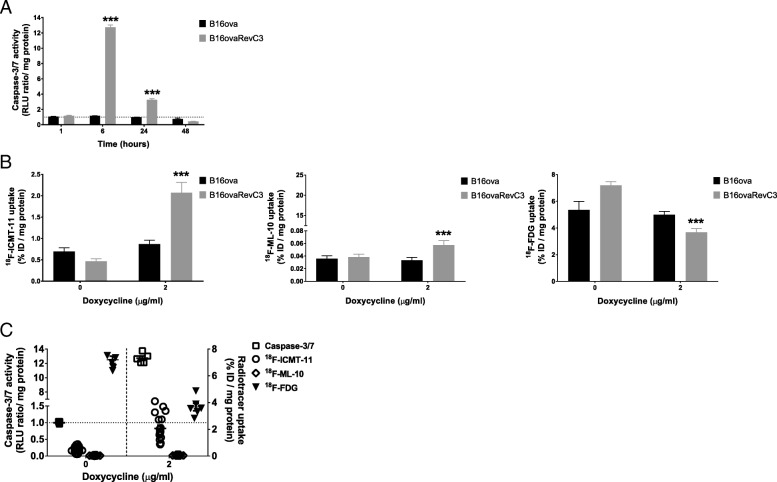
In vitro caspase activity and uptake of radiotracers in response to death-switch induction. **a** Caspase-3/7 activity in B16ova and B16ovaRevC3 cells in response to various duration of exposure to doxycycline (2 μg/ml). Significantly increased caspase activity was observed in B16ovaRevC3 cells at 6 and 24 h compared with respective controls (13- and 3-fold increase, respectively) and compared with B16ova cells treated with doxycycline (*p* < 0.001, Student’s *t* test). **b** Radiotracer uptake was evaluated following treatment with doxycycline (2 μg/ml) for 6 h. There were no significant differences in the uptake of any of the radiotracers in B16ova cells. Significantly increased uptake of ^18^F-ICMT-11 (*p* < 0.001) and ^18^F-ML-10 (*p* < 0.05), and significantly decreased uptake of ^18^F-FDG (*p* < 0.001) was observed in B16ovaRevC3 cells after death-switch induction (two-way ANOVA). Significantly higher uptake of ^18^F-ICMT-11 (*p* < 0.001) and ^18^F-ML-10 (*p* < 0.01) and significantly lower uptake of ^18^F-FDG (*p* < 0.05) were observed in B16ovaRevC3 cells compared with B16ova cells treated with doxycycline (multiple *t* tests). **c** Caspase-3/7 activity correlated with increased ^18^F-ICMT-11 and decreased ^18^F-FDG uptake in B16ovaRevC3 cells after death-switch induction. Assays were conducted in triplicate using six replicates per experiment. Data are presented as mean ± SEM. * indicates significant difference between B16ova and B16ovaRevC3 treated with doxycycline; ** indicates significant difference between doxycycline-treated B16ovaRevC3 and untreated B16ovaRevC3; *** indicates significant difference between doxycycline-treated B16ovaRevC3 and untreated B16ovaRevC3, and doxycycline-treated B16ova

We verified that caspase-3/7 induction occurs in B16ovaRevC3 cells following doxycycline exposure. Doxycycline had no significant effect on caspase-3/7 induction in B16ova cells, but a significant increase in caspase-3/7 activity was observed at 6 h (13-fold) and 24 h (3-fold) in B16ovaRevC3 cells compared with controls (Fig. 2a). Caspase-3/7 induction in B16ovaRevC3 cells was highly significant compared with B16ova cells at the 6 and 24-h time points (*p* = 0.001 and *p* < 0.001, Student’s *t* test).

### Radiotracer uptake in tumour cells

The uptake of ^18^F-ICMT-11, ^18^F-ML-10 and ^18^F-FDG was evaluated in B16ova and B16ovaRevC3 cells without and with doxycycline (0, 2 μg/ml) (Fig. 2b).

Doxycycline treatment made no difference in the uptake of any of the radiotracers in B16ova cells (Fig. 2b). However, B16ovaRevC3 cells treated with doxycycline showed significantly higher uptake of ^18^F-ICMT-11 (*p* < 0.001) and ^18^F-ML-10 (*p* < 0.05) and significantly lower uptake of ^18^F-FDG (*p* < 0.001), compared with respective controls (two-way ANOVA, Tukey’s multiple comparisons tests) (Fig. 2b).

Treatment with doxycycline resulted in significantly higher uptake of ^18^F-ICMT-11 (*p* < 0.001) and ^18^F-ML-10 (*p* < 0.01), and significantly lower uptake of ^18^F-FDG (*p* < 0.05) in B16ovaRevC3 cells compared with B16ova cells (multiple *t* tests), Fig. 2b. Caspase-3/7 activity correlated with increased ^18^F-ICMT-11 and decreased ^18^F-FDG uptake in B16ovaRevC3 cells after death-switch induction (Fig. 2c).

Significantly higher uptake of ^18^F-ICMT-11 was observed compared with ^18^F-ML-10 after death-switch induction in B16ovaRevC3 cells. The uptake of ^18^F-ICMT-11 was 36-fold higher than that of ^18^F-ML-10 with doxycycline treatment (*p* < 0.001, multiple *t* tests). Of interest, the uptake of ^18^F-ICMT-11 was 12-fold higher than that of ^18^F-ML-10 in untreated B16ovaRevC3 cells (*p* < 0.001).

### In vivo tumour growth

**Fig. 3:**
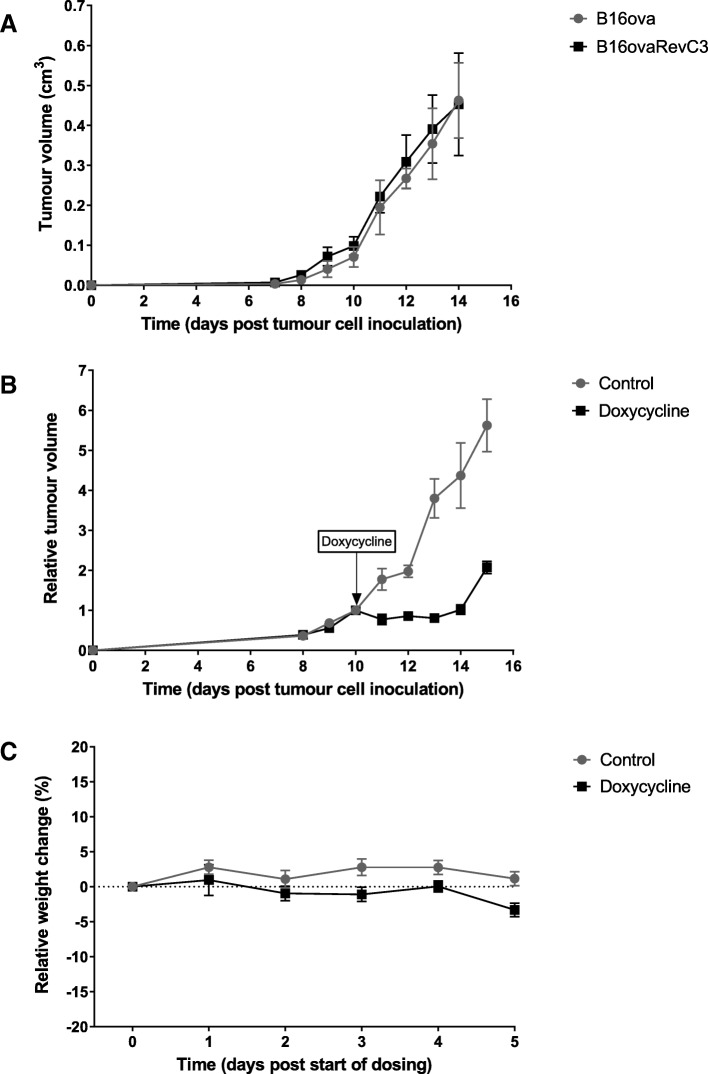
In vivo tumour growth of B16ova and B16ovaRevC3 xenografts. **a** Similar tumour growth of B16ova (*n* = 6) and B16ovaRevC3 (*n* = 6) xenografts without doxycycline administration. **b** Significantly reduced tumour growth of B16ovaRevC3 xenografts was observed in response to doxycycline compared with control B16ovaRevC3 tumours, *n* = 6 per group (at day 11, *p* < 0.01, multiple *t* tests) and persisted for the duration of doxycycline dosing (*p* < 0.001, multiple *t* tests). **c** Mouse weight relative to start of dosing with doxycycline. No observable toxicities were detected based on relative weight

Growth of B16ova and B16ovaRevC3 tumour xenografts was evaluated in vivo. Both tumour models had similar growth kinetics over time without the administration of doxycycline (Fig. 3a). However, when mice were treated with doxycycline for death-switch induction, there was a significant reduction in B16ovaRevC3 tumour growth compared with B16ovaRevC3 control tumours (starting at day 11, *p* < 0.01, multiple *t* tests), Fig. 3b. This significant reduction in tumour growth persisted for the duration of the treatment (*p* < 0.001, multiple *t* tests), Fig. 3b. There was a slight, but insignificant increase in tumour volume after the cessation of doxycycline, which is likely due to an accumulation of immune cells (as observed by IHC). A heterogeneous mix of necrosis and immune reaction, adipose formation and vesicularization within a dense stroma, were observed in tumour sections following death-switch induction.

There were no significant changes in weight between the groups (Fig. 3c).

### Biodistribution of apoptotic radiotracers

**Fig. 4:**
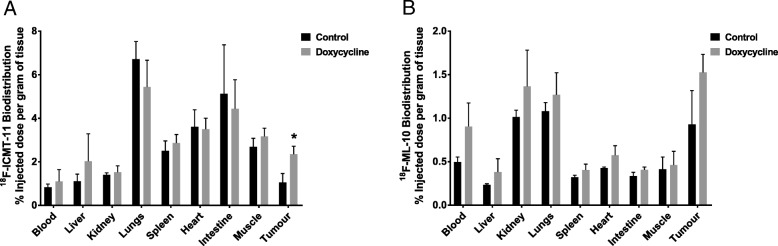
In vivo biodistribution of apoptotic radiotracers in response to death-switch induction. **a** ^18^F-ICMT-11 and **b** ^18^F-ML-10. Biodistribution of the radiotracers was assessed in the B16ovaRevC3 tumour model, 24 h after vehicle-control or doxycycline administration (*n* = 6 mice per group). Radioactivity in select tissues was measured by *ex vivo* gamma counting and biodistribution was calculated as percentage of injected dose per gram of tissue (%injected dose/g). Tumour uptake of ^18^F-ICMT-11 was significantly higher after doxycycline administration (caspase-3 activation) compared with controls (*p < 0.01, Student’s *t* test; *p* = 0.02, two-way ANOVA); however, no significant difference was observed with ^18^F-ML-10. There were no significant differences in the tissue distribution of both radiotracers between control and doxycycline-treated mice

Apoptotic radiotracer biodistribution after caspase-3 activation was evaluated in the B16ovaRevC3 tumour model by *ex vivo* gamma counting of select tissues, 24 h after vehicle or doxycycline administration (Fig. 4). Based on % injected dose/g, tumour uptake of ^18^F-ICMT-11 was 2.2-fold higher after doxycycline-induced caspase-3 activation compared with respective controls, which was significant (*p* < 0.01, Student’s *t* test; *p* = 0.02, two-way ANOVA and Sidak’s multiple comparisons tests (Fig. 4a), whereas the tumour uptake of ^18^F-ML-10 was not significantly altered after doxycycline administration (Fig. 4b). Furthermore, after caspase-3 activation, the tumour uptake of ^18^F-ICMT-11 was higher than that for ^18^F-ML-10. There were no significant differences in normal tissue distribution of the radiotracers between the vehicle-control and doxycycline-treated groups. The normal tissue distribution of ^18^F-ICMT-11 was consistent with our previous findings, showing relatively higher uptake in the small intestine [14]. Rapid hepatic ^18^F-ICMT-11 elimination could attribute to the higher intestinal uptake (the radiotracer is also eliminated via the renal clearance route). Furthermore, uptake in the small intestine could also be reflective of the prevalent physiological apoptosis of enterocytes at the villus tips and of macrophages within the intestinal lamina propria [14, 36]. Moreover, food intake can also increase radiotracer uptake in the GI tract as noted from our clinical observations [19]. As mice were fed *ad libitum*, it is likely that food intake prior imaging could have also additionally contributed to the intestinal radiotracer uptake. Apoptosis is also involved in normal lung structure and function, which can possibly contribute to some ^18^F-ICMT-11 uptake in this tissue [37]. Additional uptake could also be due to the circulating ^18^F-ICMT-11 (lungblood pool).

### PET imaging

**Fig. 5:**
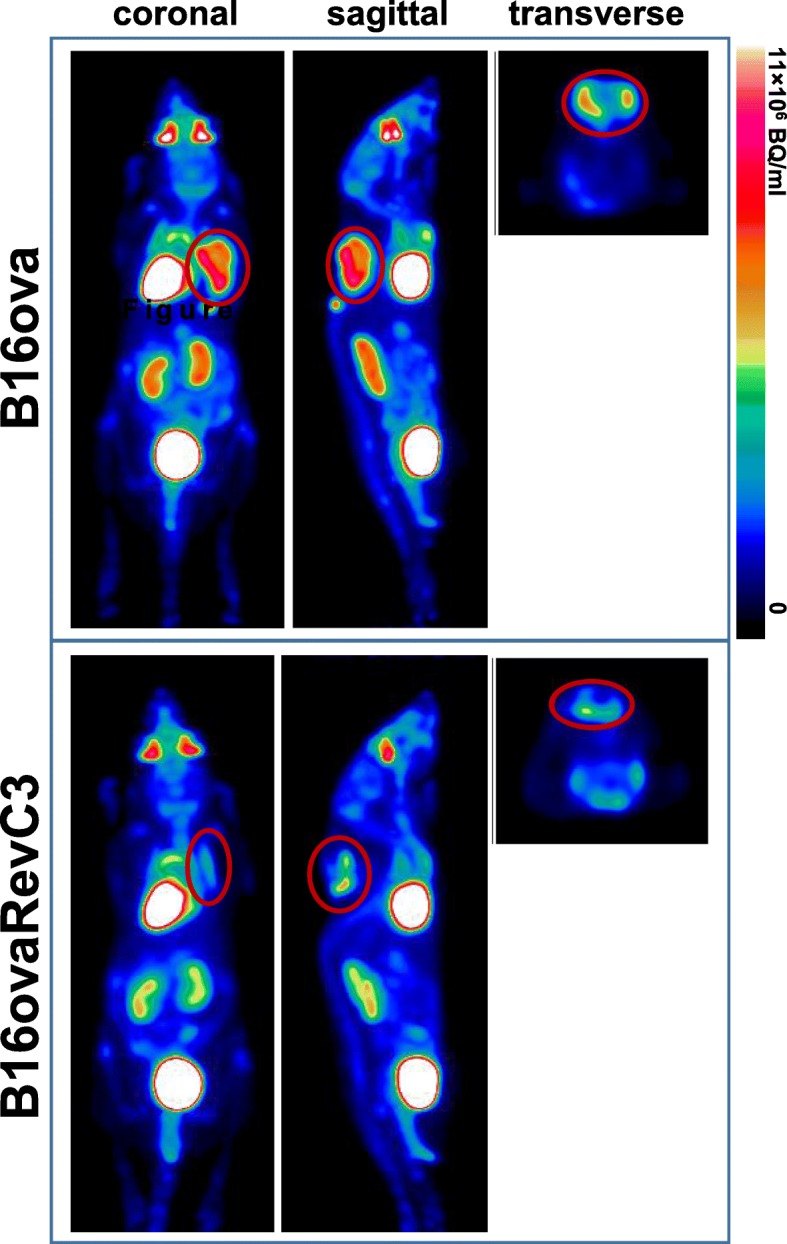
^18^F-FDG PET imaging. Representative PET images obtained with ^18^F-FDG, 24 h after doxycycline administration in mice bearing B16ova and B16ovaRevC3 tumours

We used PET imaging to assess whether caspase-3 activation can be detected using ^18^F-ICMT-11 in mice with B16ova and B16ovaRevC3 tumour xenografts at 6 and 24 h after doxycycline administration. We also conducted PET imaging using ^18^F-ML-10 and ^18^F-FDG, 24 h after vehicle-control or doxycycline administration. Representative PET images of ^18^F-ICMT-11 and ^18^F-ML-10 are displayed in Additional file 1: Figure S1, and representative images of ^18^F-FDG are displayed in Fig. 5.

### Immunohistochemistry

Detection of cleaved caspase-3 by immunohistochemistry was used to evaluate apoptosis in tumours following treatment with doxycycline for death-switch induction.

**Fig. 6:**
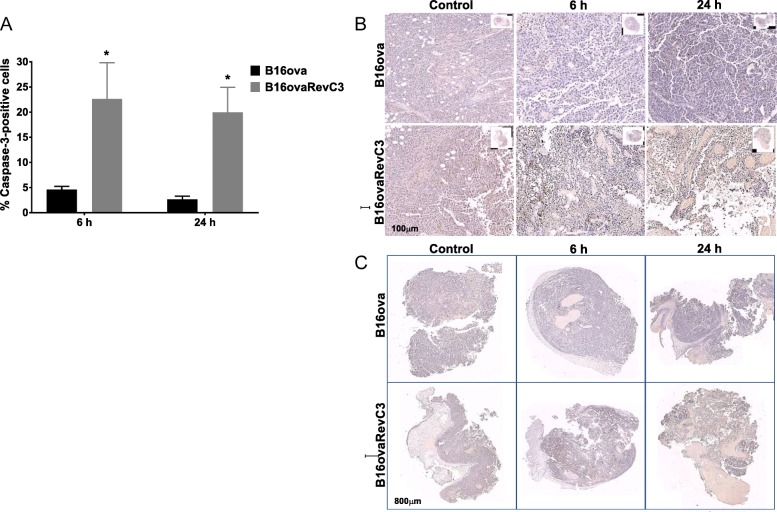
Cleaved caspase-3 expression after death-switch induction. **a** Percentage (%) caspase-3-positive cells in B16ova and B16ovaRevC3 tumour sections at 6 and 24 h after doxycycline administration. Caspase-3 expression was not detected in control B16ova or B16ovaRevC3 tumours; however, significantly increased caspase-3 expression was observed in B16ovaRevC3 tumours after death-switch induction, which was also significantly higher compared with B16ova tumours from mice treated with doxycycline (*p* < 0.05 and *p* < 0.05, respectively, multiple *t* tests). **b** Representative tumour sections stained for cleaved caspase-3 expression. **c** Representative whole tumour sections showing a heterogeneous mix of necrosis, immune reaction, patches of tumour cells positive for caspase-3 within a dense stroma, adipose formation and vesicularization following death-switch induction. * indicates significant difference between doxycycline-treated B16ovaRevC3 and control B16ovaRevC3, and doxycycline-treated B16ova

Expression of cleaved caspase-3 was negligible in tumour sections obtained from control (vehicle-treated) mice bearing B16ova or B16ovaRevC3 xenografts. However, following administration of doxycycline, the mean percentage (% ± SD) of cleaved caspase-3 expression in B16ovaRevC3 tumours was 23 ± 7 and 20 ± 5 at 6 and 24 h post-treatment, respectively, demonstrating a significant induction of apoptosis (Fig. 6a, b). This increase was 5- and 14-fold higher compared with B16ova tumours from mice treated with doxycycline (*p* < 0.05, multiple *t* tests). Of note, after death-switch induction, a heterogeneous mix of necrosis, immune reaction, patches of tumour cells positive for cleaved caspase-3 within a dense stroma, adipose formation and vesicularization was observed within B16ovaRevC3 tumour sections (Fig. 6c).

## Discussion

Several novel imaging strategies aimed at the detection and monitoring of apoptosis have been developed, and although some have progressed to clinical trials, none have been approved for routine clinical use.

We previously developed and validated a caspase-3-specific radiotracer, ^18^F-ICMT-11, for PET imaging of tumour apoptosis in several pre-clinical tumour models, and have also demonstrated safe clinical profiles of the radiotracer in phase I/II trials [14–19]. As we progress the use of ^18^F-ICMT-11 for the assessment of therapy response, it is necessary to provide validity in the context of caspase-3/7 activation, particularly where such activation is not confounded by chemotherapy. For instance, amphiphilic drugs, including doxorubicin, can induce transient channels into the cell membrane, facilitating the translocation of compounds into the intracellular membrane leaflet and hence alter cellular uptake [38]. The current study further validates ^18^F-ICMT-11 as a caspase-3-specific PET radiotracer in a doxycycline-inducible death-switch tumour model with the conditional activation of caspase-3, without the potentially complex effects of anti-cancer therapeutics [28]. We demonstrate that tumour uptake of ^18^F-ICMT-11 correlated with death-switch induction and caspase-3 activity and expression, both in vitro and in vivo, further supporting the development of ^18^F-ICMT-11 for clinical use.

We initially confirmed that apoptosis can be induced in the death-switch model in vitro. While caspase-3/7 activity was undetectable in parental cells in the presence or absence of doxycycline and in untreated death-switch cells, we confirmed that caspase-3/7 activation was robustly induced with doxycycline in B16ovaRevC3 cells. We found that in vitro, the greatest caspase-3/7 induction occurred at 6 h after treatment with doxycycline, followed by a slight decrease at 24 h, and a return to baseline levels at 48 h, which was in line with previous findings [28]. Based on these results, we investigated cellular uptake of ^18^F-ICMT-11 at the 6-h time point. We found a 4.5-fold increase in the cellular uptake of ^18^F-ICMT-11 following death-switch induction in B16ovaRevC3 cells, which correlated with elevated caspase-3/7 activity, confirming the specificity of the radiotracer for its target. The induction of apoptosis in B16ovaRevC3 cells was further supported by decreased glucose metabolism as measured by the uptake of ^18^F-FDG, which was 2-fold lower compared with controls.

By comparing ^18^F-ICMT-11 uptake to that of another apoptosis radiotracer, ^18^F-ML-10, we sought to discriminate two early features of apoptosis—specific caspase-3/7 activation and ‘apoptotic membrane imprint’; the latter proposed to be detected by ^18^F-ML-10, and comprising of acidification of the external membrane leaflet due to exposure of phosphatidylserine, permanent membrane depolarization, irreversible loss of intracellular pH control and inactivation of the phospholipid scrambling mechanism while membrane integrity is still preserved [39]. There was a 1.5-fold increase in the uptake of ^18^F-ML-10 in B16ovaRevC3 cells following death-switch induction, which was significantly lower in magnitude compared with the uptake of ^18^F-ICMT-11. Classical features of apoptosis occur within 6–12 h following death-switch induction in vitro, including membrane blebbing that has been associated with membrane depolarization, which in theory should be detected by ^18^F-ML-10 [28, 40]. The lower baseline uptake of ^18^F-ML-10, and the limited fold-change upon death-switch induction compared to ^18^F-ICMT-11 was likely reflective of (a) higher lipophilicity and bidirectional plasma membrane permeability of ^18^F-ICMT-11 compared to the peptide structure of ^18^F-ML-10 and (b) the specificity of ^18^F-ICMT-11 for caspase-3/7 induction, which is the main mechanism of apoptosis induction in the death-switch model. Another consideration is that externalization of phosphatidylserine (which leads to intracellular acidification) occurs subsequent to caspase-3/7 activation [41], which may thus limit the magnitude of selective uptake of ^18^F-ML-10 in the used model. As caspase-3/7 activation precedes phosphatidylserine exposure, imaging caspase-3/7 activity is a good strategy for the prediction of early response to treatment.

We next investigated whether these findings can be translated in vivo. We found that B16ova and B16ovaRevC3 tumours exhibited similar growth kinetics without doxycycline administration. The administration of doxycycline significantly reduced B16ovaRevC3 tumour growth, but had no effect on parental cell line xenografts, which was in line with previous findings [28]. Death-switch induction was not associated with adverse events in vivo as there were no significant changes in mouse weights. Rapid phagocytosis of apoptotic cells in this model has been demonstrated consequent to presentation of ‘danger signal’ molecules, HMGB1 and HSP90 [28], which can explain the significant early growth suppression. Given the quick phagocytosis associated with apoptosis, we wondered if these rapid tumour dynamics will result in increased apoptotic radiotracer uptake and permit in vivo imaging. Thus, we evaluated whether tumour distribution of the apoptotic radiotracers, ^18^F-ICMT-11 and ^18^F-ML-18, was altered following death-switch induction in B16ovaRevC3 tumours. Based on *ex vivo* gamma counting, the tumour uptake of ^18^F-ICMT-11 was significantly higher after caspase-3 activation (death-switch induction), reflecting the specificity of the radiotracer for the target, whereas no significant change was observed for ^18^F-ML-10. Similar to our in vitro findings, in vivo ^18^F-ICMT-11 tumour distribution was significantly higher compared with ^18^F-ML-10, further supporting the specificity of ^18^F-ICMT-11 for activated caspase-3, which is the main mechanism of apoptosis induction in the death-switch model.

Of note, a recent study demonstrated an increased uptake of ^18^F-ML-18 after doxorubicin administration in head and neck tumour xenografts at 3 and 7 days post-treatment, but with minimal change at day 1 [33]. However, the authors did not correlate these findings to caspase-3 activity; instead, the TUNEL assay was used for the assessment of apoptosis, which is a measure of the apoptotic process at a later stage. Another pre-clinical study showed increased ^18^F-ML-10 uptake in nasopharyngeal carcinoma at 24 and 48 h after combined radiotherapy and cetuximab treatment, which correlated with increased TUNEL staining [34]. Ionising radiation can cause cell death via numerous mechanisms, including apoptosis, necrosis, mitotic catastrophe and senescence, which differ from the mechanism of induction in the death-switch tumour model, which could allow for detection with ^18^F-ML-10. Nonetheless, some of these can alter membrane permeability and integrity, and hence radiotracer distribution.

The fold-difference in the in vivo uptake of ^18^F-ICMT-11 after death-switch induction was lower than that seen in vitro (2.2- vs 4.5-fold, respectively), which was anticipated, given that in vivo evolution of response is expected to be different from in vitro dynamics. Potential reasons for this are as follows: (a) the possibility that changes induced within the tumour microenvironment after death-switch induction could have altered the tumour uptake of the radiotracer, hence not fully reflecting the proportion of tumour cells undergoing apoptosis. For example, a heterogeneous mix of necrosis, immune reaction, patches of tumour cells undergoing apoptosis within a dense stroma, adipose formation and vesicularization was observed within these tumours after death-switch induction. It is possible that these transformations can lead to the compression of blood vessels and hence limit the amount of ^18^F-ICMT-11 reaching its target in tumour cells; (b) *Melis* et al. found that after death-switch induction, tumours undergo rapid caspase-dependent cell death, along with the release of aforementioned endogenous danger signals and quick progression towards secondary necrosis with evidence of an immune response [28], which is in support of our observations. Moreover, the difference between the in vivo and in vitro findings might be a result of the rapid clearance of apoptotic cells by host phagocytes or other confounding factors such as extracellular acidification occurring in vivo. Furthermore, the methods used for the evaluation of caspase-3 in vitro and in vivo were different—the Caspase-Glo activity assay and IHC for cleaved caspase-3, respectively.

We also investigated whether caspase-3 activation can be detected by PET imaging using ^18^F-ICMT-11 in a time-course study; however, the data obtained from these tumour models were too subjective for quantification. Nonetheless, PET imaging showed that caspase-3 activation can be detected using ^18^F-ICMT-11 in B16ovaRevC3 tumours, 6 and 24 h after death-switch induction, which was also accompanied by significantly increased expression of cleaved capase-3 in tumour sections, as demonstrated by IHC, confirming the induction of apoptosis in these tumours.

Interestingly, PET imaging revealed a decrease in ^18^F-FDG signal following death-switch induction, suggesting a decrease in glucose metabolism or tumour growth. A significant reduction in tumour volume was also observed, but only at 24 h, corroborating that tumour volume measurements alone are not sufficient for the prediction of response to therapy. These findings support the use of molecular imaging to predict early response to therapy since the induction of apoptosis was detected by ^18^F-ICMT-11 PET imaging as early as 6 h, prior a macroscopically measurable response to treatment.

Beyond apoptosis imaging, the use of metabolism radiotracers to assess response to therapy is a routine clinical practice. Besides the gold-standard ^18^F-FDG, ^18^F-FLT is another radiotracer used for the assessment of tumour cell metabolism; however, in contrast to ^18^F-FDG, ^18^F-FLT appears not to accumulate in inflammatory processes [42, 43]. While ^18^F-FDG and ^18^F-FLT are useful tools for the detection of changes in energy metabolism and cell proliferation, the use of these radiotracers for prediction of early response to treatment has provided variable results [44–48]. Therefore, detection of early efficacy remains a challenge, and the addition of ^18^F-ICMT-11 to routine PET imaging can be beneficial in stratifying patients into responders and non-responders. Indeed, we have previously assessed ^18^F-ICMT-11 in numerous pre-clinical tumour models and have demonstrated added value of ^18^F-ICMT-11 to ^18^F-FDG, ^18^F-FLT and diffusion-weighted MRI [16, 49].

## Conclusion

In conclusion, we demonstrate that ^18^F-ICMT-11 can detect caspase-3 activation in a doxycycline-inducible death-switch model, which further validates the specificity of the tracer without the confounding effects of anti-cancer therapeutics. These findings, along with previous evidence, verify that ^18^F-ICMT-11 PET imaging permits the real-time assessment of caspase-3 activation over time, and thus further support the development of this radiotracer for clinical use to monitor tumour apoptosis in response to treatment. Timing and image analysis strategies should be optimized based on the type of tumour and treatment regimen, and taking into consideration the clinical heterogeneity and dynamics of apoptosis for ultimate assessment of treatment outcome.

## Additional file

Additional file 1: **Figure S1.** PET imaging with apoptotic radiotracers. Representative PET images with (A) ^18^F-ICMT-11 at 6 and 24 h and (B) ^18^F-ML-10 at 24 h, after doxycycline administration in mice bearing B16ova and B16ovaRevC3 tumours. (PDF 560 kb)

## References

[1] Reed JC (2002). Apoptosis-based therapies. Nat Rev Drug Discov.

[2] Hanahan D, Weinberg RA (2000). The hallmarks of cancer. Cell.

[3] Hanahan D, Weinberg RA (2011). Hallmarks of cancer: the next generation. Cell.

[4] Okada H, Mak TW (2004). Pathways of apoptotic and non-apoptotic death in tumour cells. Nat Rev Cancer.

[5] Dimri GP (2005). What has senescence got to do with cancer?. Cancer Cell.

[6] Degterev A, Boyce M, Yuan J (2003). A decade of caspases. Oncogene.

[7] Nicholson DW, Thornberry NA (1997). Caspases: killer proteases. Trends Biochem Sci.

[8] Pop C, Salvesen GS (2009). Human caspases: activation, specificity, and regulation. J Biol Chem.

[9] Taylor RC, Cullen SP, Martin SJ (2008). Apoptosis: controlled demolition at the cellular level. Nat Rev Mol Cell Biol.

[10] Lakhani SA, Masud A, Kuida K, Porter GA, Booth CJ, Mehal WZ (2006). Caspases 3 and 7: key mediators of mitochondrial events of apoptosis. Science (New York, NY).

[11] Porter AG, Jänicke RU (1999). Emerging roles of caspase-3 in apoptosis. Cell Death Differ.

[12] Lee D, Long SA, Adams JL, Chan G, Vaidya KS, Francis TA (2000). Potent and selective nonpeptide inhibitors of caspases 3 and 7 inhibit apoptosis and maintain cell functionality. J Biol Chem.

[13] Chu W, Zhang J, Zeng C, Rothfuss J, Tu Z, Chu Y (2005). N-Benzylisatin sulfonamide analogues as potent caspase-3 inhibitors: synthesis, in vitro activity, and molecular modeling studies. J Med Chem.

[14] Nguyen Q-D, Smith G, Glaser M, Perumal M, Årstad E, Aboagye EO (2009). Positron emission tomography imaging of drug-induced tumor apoptosis with a caspase-3/7 specific [(18)F]-labeled isatin sulfonamide. Proc Natl Acad Sci U S A.

[15] Glaser M, Goggi J, Smith G, Morrison M, Luthra SK, Robins E (2011). Improved radiosynthesis of the apoptosis marker 18F-ICMT11 including biological evaluation. Bioorg Med Chem Lett.

[16] Nguyen Q-D, Lavdas I, Gubbins J, Smith G, Fortt R, Carroll LS (2013). Temporal and spatial evolution of therapy-induced tumor apoptosis detected by caspase-3–selective molecular imaging. Clin Cancer Res.

[17] Witney TH, Fortt RR, Aboagye EO (2014). Preclinical assessment of carboplatin treatment efficacy in lung cancer by (18)F-ICMT-11-positron emission tomography. PLoS One.

[18] Nguyen Q-D, Challapalli A, Smith G, Fortt R, Aboagye EO (2012). Imaging apoptosis with positron emission tomography: ‘bench to bedside’ development of the caspase-3/7 specific radiotracer [18F]ICMT-11. Eur J Cancer.

[19] Challapalli A, Kenny LM, Hallett WA, Kozlowski K, Tomasi G, Gudi M (2013). 18F-ICMT-11, a caspase-3–specific PET tracer for apoptosis: biodistribution and radiation dosimetry. J Nucl Med.

[20] Vermes I, Haanen C, Steffens-Nakken H, Reutellingsperger C (1995). A novel assay for apoptosis flow cytometric detection of phosphatidylserine expression on early apoptotic cells using fluorescein labelled Annexin V. J Immunol Methods.

[21] Koopman G, Reutelingsperger CP, Kuijten GA, Keehnen RM, Pals ST, van Oers MH (1994). Annexin V for flow cytometric detection of phosphatidylserine expression on B cells undergoing apoptosis. Blood.

[22] Zhu X, Li Z, Zhao M (2007). Imaging acute cardiac cell death: temporal and spatial distribution of 99mTc-labeled C2A in the area at risk after myocardial ischemia and reperfusion. J Nucl Med.

[23] Witney TH, Hoehne A, Reeves RE, Ilovich O, Namavari M, Shen B (2015). A systematic comparison of (18)F-C-SNAT to established radiotracer imaging agents for the detection of tumor response to treatment. Clin Cancer Res.

[24] Madar I, Huang Y, Ravert H, Dalrymple SL, Davidson NE, Isaacs JT (2009). Detection and quantification of the evolution dynamics of apoptosis using the PET voltage sensor 18F-fluorobenzyl triphenyl phosphonium. J Nucl Med.

[25] Su H, Chen G, Gangadharmath U, Gomez LF, Liang Q, Mu F (2013). Evaluation of [18F]-CP18 as a PET imaging tracer for apoptosis. Mol Imaging Biol.

[26] Reshef A, Shirvan A, Akselrod-Ballin A, Wall A, Ziv I (2010). Small-molecule biomarkers for clinical PET imaging of apoptosis. J Nucl Med.

[27] Smith G, Carroll L, Aboagye EO (2012). New frontiers in the design and synthesis of imaging probes for PET oncology: current challenges and future directions. Mol Imaging Biol.

[28] Melis MH, Simpson KL, Dovedi SJ, Welman A, MacFarlane M, Dive C (2013). Sustained tumour eradication after induced caspase-3 activation and synchronous tumour apoptosis requires an intact host immune response. Cell Death Differ.

[29] Dewkar GK, Sundaresan G, Lamichhane N, Hirsch J, Thadigiri C, Collier T (2013). Microfluidic radiosynthesis and biodistribution of [18F] 2-(5-fluoro-pentyl)-2-methyl malonic acid. J Label Compd Radiopharm.

[30] Fortt R, Smith G, Awais RO, Luthra SK, Aboagye EO (2012). Automated GMP synthesis of [18F]ICMT-11 for in vivo imaging of caspase-3 activity. Nucl Med Biol.

[31] Workman P, Aboagye EO, Balkwill F, Balmain A, Bruder G, Chaplin DJ (2010). Guidelines for the welfare and use of animals in cancer research. Br J Cancer.

[32] Zeidner NS, Brandt KS, Dadey E, Dolan MC, Happ C, Piesman J (2004). Sustained-release formulation of doxycycline hyclate for prophylaxis of tick bite infection in a murine model of Lyme borreliosis. Antimicrob Agents Chemother.

[33] Demirci E, Ahmed R, Ocak M, Latoche J, Radelet A, DeBlasio N (2017). Preclinical evaluation of (18)F-ML-10 to determine timing of apoptotic response to chemotherapy in solid tumors. Mol Imaging.

[34] Gu B, Liu S, Sun Y, Zhang J, Zhang Y, Xu X, et al. Predictive value of [18F]ML-10 PET/CT in early response evaluation of combination radiotherapy with cetuximab on nasopharyngeal carcinoma. Mol Imaging Biol. 2018. 10.1007/s11307-018-1277-9. 30218389

[35] Bankhead P, Loughrey MB, Fernández JA, Dombrowski Y, McArt DG, Dunne PD (2017). QuPath: open source software for digital pathology image analysis. Sci Rep.

[36] Aschoff A, Günther E, Jirikowski G (2000). Tissue transglutaminase in the small intestine of the mouse as a marker for apoptotic cells. Colocalization with DNA fragmentation.

[37] Henson PM, Tuder RM (2008). Apoptosis in the lung: induction, clearance and detection. Am J Physiol Lung Cell Mol Physiol.

[38] van Hell AJ, Melo MN, van Blitterswijk WJ, Gueth DM, Braumuller TM, Pedrosa LRC (2013). Defined lipid analogues induce transient channels to facilitate drug-membrane traversal and circumvent cancer therapy resistance. Sci Rep.

[39] Cohen A, Shirvan A, Levin G, Grimberg H, Reshef A, Ziv I (2009). From the Gla domain to a novel small-molecule detector of apoptosis. Cell Res.

[40] Bratton DL, Fadok VA, Richter DA, Kailey JM, Guthrie LA, Henson PM (1997). Appearance of phosphatidylserine on apoptotic cells requires calcium-mediated nonspecific flip-flop and is enhanced by loss of the aminophospholipid translocase. J Biol Chem.

[41] Birge RB, Boeltz S, Kumar S, Carlson J, Wanderley J, Calianese D (2016). Phosphatidylserine is a global immunosuppressive signal in efferocytosis, infectious disease, and cancer. Cell Death Differ.

[42] van Waarde A, Cobben DCP, Suurmeijer AJH, Maas B, Vaalburg W, de Vries EFJ (2004). Selectivity of 18F-FLT and 18F-FDG for differentiating tumor from inflammation in a rodent model. J Nucl Med.

[43] Bollineni VR, Kramer GM, Jansma EP, Liu Y, Oyen WJG (2016). A systematic review on [18F]FLT-PET uptake as a measure of treatment response in cancer patients. Eur J Cancer.

[44] Jensen MM, Kjaer A (2015). Monitoring of anti-cancer treatment with (18)F-FDG and (18)F-FLT PET: a comprehensive review of pre-clinical studies. Am J Nucl Med Mol Imaging.

[45] Schelhaas S, Heinzmann K, Bollineni VR, Kramer GM, Liu Y, Waterton JC (2017). Preclinical applications of 3′-deoxy-3′-[(18)F]fluorothymidine in oncology - a systematic review. Theranostics.

[46] Crandall JP, Tahari AK, Juergens RA, Brahmer JR, Rudin CM, Esposito G (2017). A comparison of FLT to FDG PET/CT in the early assessment of chemotherapy response in stages IB–IIIA resectable NSCLC. EJNMMI Res.

[47] Gerbaudo VH, Killoran JH, Kim CK, Hornick JL, Nowak JA, Enzinger PC (2018). Pilot study of serial FLT and FDG-PET/CT imaging to monitor response to neoadjuvant chemoradiotherapy of esophageal adenocarcinoma: correlation with histopathologic response. Ann Nucl Med.

[48] Kenny L, Coombes RC, Vigushin DM, Al-Nahhas A, Shousha S, Aboagye EO (2007). Imaging early changes in proliferation at 1 week post chemotherapy: a pilot study in breast cancer patients with 3′-deoxy-3′-[18F]fluorothymidine positron emission tomography. Eur J Nucl Med Mol Imaging.

[49] Heinzmann K, Nguyen Q-D, Honess DJ, Smith D-M, Stribbling S, Brickute D, et al. Depicting changes in tumor biology in response to cetuximab mono- or combination therapy by apoptosis and proliferation imaging using 18F-ICMT-11 and 3′-Deoxy-3′-[18F]Fluorothymidine (18F-FLT) PET. J Nucl Med. 2018;59(10):1558–65. 10.2967/jnumed.118.209304 PMC6167530 29794225

