# Supplementary figures and images for: Evaluation of apoptosis imaging biomarkers in a genetic model of cell death
Source: EJNMMI Res. 2019 Feb 19;9:18. doi: 10.1186/s13550-019-0487-8 (PMC6381199; doi:10.1186/s13550-019-0487-8)

Figure S1

A

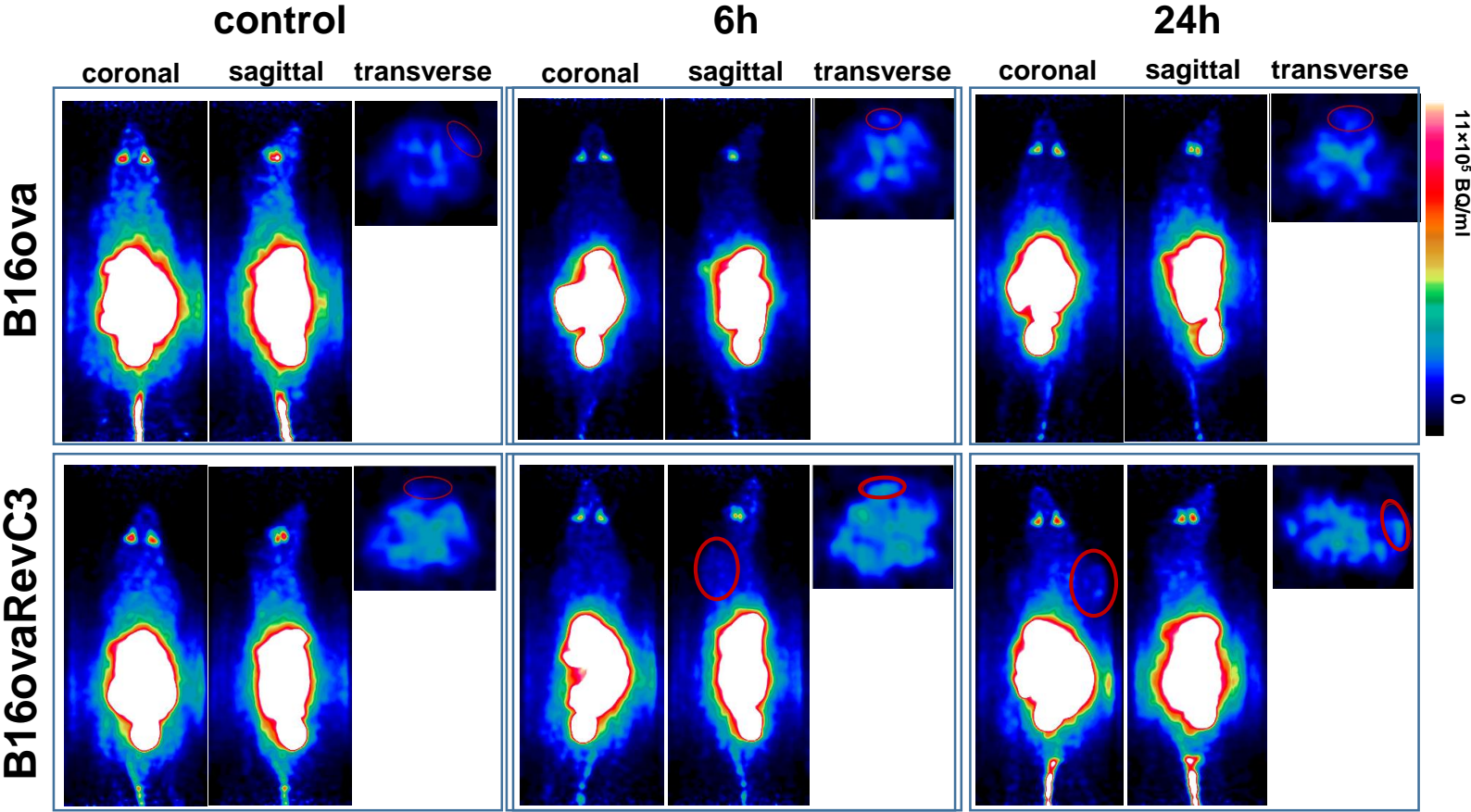

B

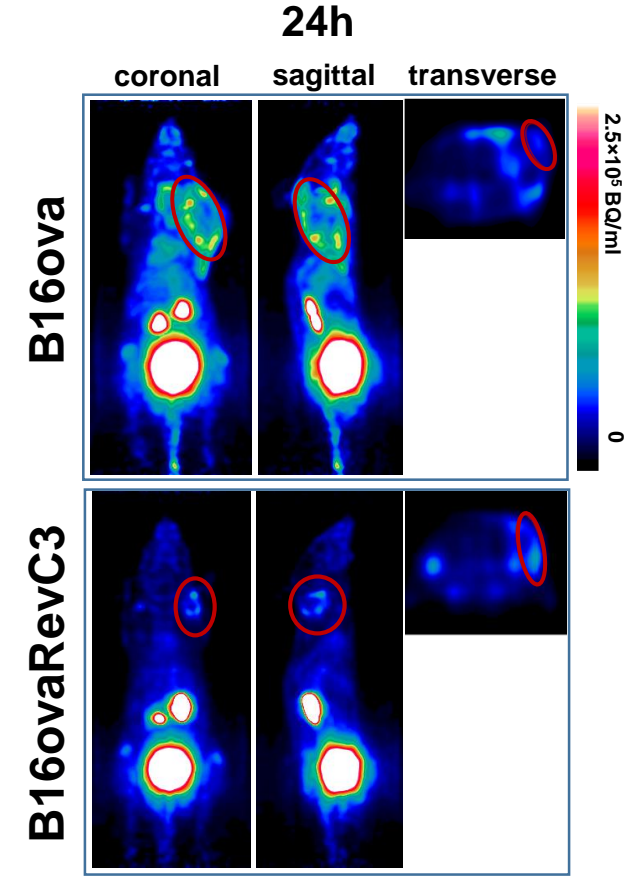

Supplement: Supplementary file 1 — Figure S1. PET imaging with apoptotic radiotracers. Representative PET images with (A) 18F-ICMT-11 at 6 and 24 h and (B) 18F-ML-10 at 24 h, after doxycycline administration in mice bearing B16ova and B16ovaRevC3 tumours. (PDF 560 kb) [file 13550_2019_487_MOESM1_ESM.pdf]
